# Association of dietary behaviour and depression among adolescents in Malaysia: a cross-sectional study

**DOI:** 10.1186/s41043-023-00480-5

**Published:** 2023-11-28

**Authors:** Norhafizah Sahril, Muhammad Azri Adam Adnan, Muhamad Khairul Nazrin Khalil, Yee Mang Chan, Kishwen Kanna Yoga Ratnam, Wai Kent Lai, Noor Ani Ahmad

**Affiliations:** Institute for Public Health, National Institutes of Health, Ministry of Health Malaysia, Block B5, No 1, Jalan Setia Murni U13/52, Seksyen U13, Setia Alam, 40170 Shah Alam, Malaysia

**Keywords:** Depression, Adolescents, Dietary behaviour, National School-Based Health Survey

## Abstract

**Background:**

Depression is on the rise and has become a significant concern for public health. Limited research has explored the connection between dietary patterns and depression. This investigation aimed to examine how dietary behaviours relate to depression among adolescents attending school in Malaysia.

**Methods:**

The study utilized data from the National School-Based Health Survey 2022, a nationwide survey involving school-going adolescents aged 13–17, with a total of 33,523 participants from 239 schools. To assess depression, the study employed the Patient Health Questionnaire (PHQ-9), considering a score of 10 or higher as indicative of depression. Dietary behaviours were assessed using a validated self-administered questionnaire adapted from the World Health Organization's Global School-based Student Health Survey. Descriptive analysis and complex sample logistic regression were carried out using SPSS version 26.0.

**Results:**

The study revealed a 26.9% overall prevalence of depression, with higher risks among female adolescents (AOR 2.71, 95% CI 2.51, 2.92) and those in higher grades. Malays (AOR 1.71, 95% CI 1.41, 2.07), Other Bumiputeras (AOR 1.69, 95% CI 1.32, 2.17), and Others (AOR 1.63, 95% CI 1.16, 2.30) had elevated odds of depression compared to Indians. Healthy dietary habits, like consuming less than two servings of fruits daily, correlated with depression (AOR 1.44 95% CI 1.35, 1.54). Additionally, unhealthy dietary behaviours such as fast food consumption (AOR 1.73 [95% CI 1.55, 1.93]) and carbonated soft drink intake (AOR 1.59 [95% CI 1.48, 1.70]) were positively associated with depression.

**Conclusions:**

One out of every four Malaysian adolescents was identified to experience depression. Depression was linked to several factors, such as being female, belonging to higher grade levels, identifying as Malays, Other Bumiputeras, or belonging to other ethnicities, and participating in the consumption of fast food, carbonated soft drinks, and fruits. To address these observations, it is crucial to formulate effective public health programmes that prioritize adolescent mental health and encourage the adoption of healthy eating habits.

## Background

Depression is a common mental health problem that affects adolescents all around the world. It can show as a variety of symptoms such as feeling of sadness, guilt, low self-esteem, unhappiness, and dissatisfaction with their surroundings [1]. Furthermore, depression can cause sleep difficulties, lack of appetite, decreased energy levels, and a heightened sense of hopelessness, which can lead to suicidal ideation [2]. Globally, around 7.4% of adolescents in the 10 to 14-year age group experience depression [3]. According to the World Health Organization (WHO) 2021, depression affects approximately 1.1% of adolescents aged 10 to 14 years and 2.8% of those aged 15–19 years [4]. In Malaysia, data from the National Health and Morbidity Survey (NHMS) in 2019 revealed a 2.1% prevalence of depression among individuals aged 15–19 years old [5].

Recent and emerging research has shed light on a profound and meaningful correlation between an individual's dietary preferences and their mental health status, especially concerning conditions like depression [6, 7]. The maintenance of a well-balanced and nourishing dietary routine is intrinsically tied to the enhancement of one's mental well-being. Specific components in our diets have the potential to wield substantial influence over metabolic processes, contributing to the intricate and multifaceted interaction between diet and mood [8].

Additionally, there is a growing acknowledgment of the pivotal role that eating habits play in shaping an individual's mental well-being [9]. It has come to our attention that adolescents who adhere to unhealthy dietary patterns, such as excessive consumption of full-fat dairy products, saturated fatty acids (SFAs), red and processed meats, and refined sugars, are more prone to developing depression, enduring persistent low spirits, and grappling with anxiety [7, 10–12]. Conversely, embracing healthier eating habits has the capacity to reduce the likelihood of experiencing symptoms of depression. Moreover, unfavourable eating habits, like a deficiency of fruits and vegetables, have been associated with feelings of sorrow or despair, contemplation of suicide, and even actual suicide attempts among adolescents [13–16].

Furthermore, compelling and substantial evidence vigorously supports the idea that avoiding processed foods and adhering to wholesome dietary patterns can serve as effective preventive measures against the onset of depression [6]. Consequently, a concept has emerged indicating that a variety of poor dietary choices may be closely linked to an increased risk of depression.

Adolescence represents a pivotal phase of development characterized by significant physical, emotional, and social transformations, along with the adoption of enduring eating habits. The evolving dietary choices and patterns across diverse cultural contexts give rise to genuine concerns regarding their potential impact on mental health, particularly with regard to depression. It is imperative to thoroughly explore the intricate connections between dietary behaviours and depression within this adolescent population. Such an inquiry lays the groundwork for effective strategies aimed at both preventing and addressing mental health issues, with the potential to enhance mental well-being and reduce the risk of depression. To summarize, the body of research in this specialized field in Malaysia remains somewhat limited, and the precise nature of the relationship between dietary behaviours and depression remains somewhat unclear. Consequently, the present study was conceived and conducted with the primary objective of elucidating the link between depression and dietary behaviours among Malaysian school-going adolescents.

## Methods

### Study design and sampling

Data for this study were derived from the National School-Based Health Survey 2022, which constitutes a cross-sectional examination encompassing secondary school students aged 13 to 17 years in Malaysia. This survey was conducted during the period of June to July 2022. To gather these data, a multistage stratified cluster sampling approach was employed, consisting of two distinct stages. In the initial stage, the selection of secondary schools from the comprehensive list of eligible schools in Malaysia was executed. A total of 240 schools were randomly chosen, with selection proportional to enrolment (PPS) across Forms 1, 2, 3, 4, and 5. In each state, 16 schools were selected, with the exception of two smaller federal territories, Labuan and Putrajaya, where eight schools were chosen in each. Subsequently, the second stage involved the selection of classes. All classes spanning Forms 1, 2, 3, 4, and 5 were incorporated into the sampling frame. The selection of classes from each chosen school was achieved using systematic probability sampling, with a random starting point. All students within the selected classes were invited to participate in the survey, provided they had obtained written parental consent. The criteria for inclusion encompassed students aged between 13 and 17 years old. The exclusion criteria were absentees on the days when the study was carried out and those who did not obtain parental consent to participate. Trained researchers provided these students with information regarding the survey and instructions for completing the self-administered questionnaires. Ethical approvals for this study were duly secured from the Medical Research and Ethics Committee (MREC), Ministry of Health Malaysia (NMRR-21–157-58,261). The permission to conduct the study was obtained from the Ministry of Education at the national, state and school levels. The completed methods of the National School-Based Health Survey 2022 can be found elsewhere [17].

## Measures

### Depression

The variable under examination, which is depression, was assessed using the Patient Health Questionnaire-9 (PHQ-9). This self-report tool comprises nine questions aligned with the nine DSM-IV criteria used for diagnosing depression. It is employed to ascertain the presence or absence of depression. In this study, the validated Malay version of the PHQ-9 was utilized to identify depression [18]. The original English version, authored by Kroenke, Spitzer, and Williams in 2001, served as the basis for this instrument [19]. The questionnaire inquiries about symptoms experienced over the two weeks preceding its completion. Each of the nine items offers four response options ("not at all”, "several days”, "more than half the days”, and "nearly every day"), with corresponding scores ranging from 0 (indicating no occurrence) to 3 (indicating almost daily occurrence), resulting in a total score ranging from 0 to 27. Respondents scoring 10 or higher were classified as individuals experiencing depression [18].

### Independent variables

The National School-Based Health Survey 2022 employed a self-administered questionnaire, which was adapted from the World Health Organization's Global School-based Student Health Survey and was available in two languages. In this study, independent variables encompassed socio-demographic factors and dietary behaviours. Socio-demographic variables consisted of sex, grade level (form), and ethnicity. On the flip side, the variables related to dietary behaviours encompassed the consumption of fruits, vegetables, fast food, and carbonated soft drinks. Fruit consumption was assessed by whether participants had consumed fruits at least twice daily in the preceding 30 days, encompassing all varieties of fruits. Vegetable consumption was gauged by whether participants had consumed vegetables at least three times daily in the past 30 days. Adequate consumption of fruits and vegetables was determined based on the 2021 Malaysia Dietary Guidelines, which recommend a daily intake of a minimum of two servings of fruits and three servings of vegetables [20]. Carbonated soft drink consumption was categorized as having occurred at least once daily within the past 30 days. Meanwhile, fast food consumption was evaluated by examining the frequency of consuming food from fast food outlets, with a minimum threshold of three days within the preceding seven days.

### Data analysis

Data were analysed using SPSS version 26 (SPSS IBM, New York, USA). Data were weighted to account for the complex study design and nonresponse rate. Descriptive statistics were used to describe the socio-demographic characteristics and prevalence of depressive symptoms among adolescents. Factors associated with depression were analysed using multivariate logistic regression analysis. All statistical analyses were carried out at a 95% confidence interval or *P value* < 0.05.

## Results

A total of 33,523 adolescents attending school took part in this study. The characteristics of the respondents revealed that a majority of the adolescents were female (53.8%) and of Malays ethnic (69.0%). Participation was fairly evenly distributed among various grade levels (Forms). When it comes to their dietary habits, 37.8% of the adolescents indicated they had consumed fruits at least twice daily, 26.2% reported eating vegetables at least three times daily, and 31.7% had consumed carbonated soft drinks at least once daily in the 30 days preceding the survey. Furthermore, approximately 10.9% of adolescents stated that they had eaten fast food on a minimum of three occasions during the seven days leading up to the survey (Table 1).

**Table 1:** Socio-demographic characteristics (N = 33,523)

Socio-demographic characteristic	*n*	Percentage (%)
*Sex*		
Female	18,030	53.8
Male	15,493	46.2
*Form*		
1	7216	21.5
2	6902	20.6
3	6460	19.3
4	6756	20.2
5	6189	18.5
*Ethnicity*		
Malay	23,125	69.0
Chinese	5085	15.2
Indian	1556	4.6
Other Bumiputeras	2963	8.8
Others	794	2.4
*Consuming fast food*		
Less than 3 days per week	29,846	89.1
At least 3 days per week	3648	10.9
*Carbonated soft drinks consumption*		
Less than 1 time per day	22,879	68.3
At least 1 time per day	10,614	31.7
*Fruits intake*		
Less than 2 servings per day	20,841	62.2
At least 2 servings per day	12,655	37.8
*Vegetable intakes)*		
Less than 3 servings per day	24,732	73.8
At least 3 servings per day	8762	26.2

The prevalence of depression among Malaysian adolescents was recorded at 26.9% (95% CI 25.84, 27.96), impacting a total of 556,498 adolescents. It's worth noting that the prevalence of depression was notably higher among females, with a rate of 36.1% (95% CI 34.58, 37.68), in contrast to males, where it was 17.7% (95% CI 16.69, 18.67). Furthermore, depression rates were significantly elevated among those who had consumed fast food on at least three occasions, reaching 39.0% (95% CI 36.49, 41.65), as well as among those who consumed carbonated soft drinks at least once daily, with a prevalence of 32.4% (95% CI 30.92, 33.93). Additionally, adolescents who reported consuming fruit less than twice daily exhibited a significantly higher prevalence of depression, standing at 28.8% (95% CI 27.68, 30.03) (Table 2).

**Table 2:** Prevalence of depression by socio-demographic and dietary behaviour (N = 9,103)

Socio-demographic characteristic	Count (*n*)	Estimated population	Prevalence (%)	95% confidence interval	
				Lower	Upper
Overall	9103	556,498	26.9	25.84	27.96
*Sex*					
Female	6421	373,651	36.1	34.58	37.68
Male	2682	182,847	17.7	16.69	18.67
*Form*					
1	1704	101,399	22.6	21.18	24.02
2	1882	117,524	27.2	25.58	28.83
3	1802	112,513	27.0	25.08	28.94
4	1882	110,606	28.4	26.61	30.16
5	1833	114,456	30.1	28.22	31.95
*Ethnicity*					
Malay	6504	373,425	28.7	27.46	29.89
Chinese	1065	77,209	20.6	18.31	23.05
Indian	328	24,848	20.2	17.59	23.04
Other Bumiputeras	977	67,843	30.5	27.07	34.13
Others	229	13,172	28.6	23.44	34.38
*Consuming fast food*					
Less than 3 days per week	7688	471,121	25.5	24.44	26.51
At least 3 days per week	1409	85,006	39.0	36.49	41.65
*Carbonated soft drinks consumption*					
Less than 1 time per day	5646	339,145	24.2	23.19	25.35
At least 1 time per day	3451	216,943	32.4	30.92	33.93
*Fruits intake*					
Less than 2 servings per day	6091	373,942	28.8	27.68	30.03
At least 2 servings per day	3006	182,059	23.6	22.41	24.88
*Vegetable intakes*					
Less than 3 servings per day	6886	415,559	27.5	26.40	28.71
At least 3 servings per day	2209	140,366	25.1	23.70	26.55

The results from the multiple logistic regression analysis revealed several important findings. Female adolescents (Adjusted Odd Ratio (AOR): 2.71, 95% Confidence Interval (CI): 2.51, 2.92) and those in higher academic forms had a higher odd of experiencing depression. In terms of ethnicity, Malay adolescents (AOR 1.71, 95% CI 1.41, 2.07), Other Bumiputeras (AOR 1.69, 95% CI 1.32, 2.17), and Others (AOR 1.63, 95% CI 1.16, 2.30) exhibited a higher likelihood of experiencing depression. Additionally, this study revealed a connection between unhealthy dietary behaviours, such as the intake of fast food at least three days a week (AOR 1.73, 95% CI 1.55, 1.93), and carbonated soft drinks at least once a day (AOR 1.59, 95% CI 1.48, 1.70), and the likelihood of experiencing depression. Furthermore, an observed association was identified between healthy dietary behaviours, specifically consuming less than two servings of fruit per day (AOR 1.44, 95% CI 1.35, 1.54), and depression (Table 3).

**Table 3:** Factor associated with depression among school-going adolescents in Malaysia

Socio-demographic characteristic	Crude OR			*p* value	Adjusted OR			*p* value
	Exp (B)	95% confidence interval			Exp(B)	95% confidence interval		
		Lower	Upper			Lower	Upper	
*Sex*								
Male	1.00				1.00			
Female	2.64	2.45	2.84	< 0.001	2.71	2.51	2.92	< 0.001
*Form*								
1	1.00				1.00			
2	1.28	1.16	1.42	< 0.001	1.29	1.16	1.41	< 0.001
3	1.27	1.13	1.42	< 0.001	1.27	1.13	1.43	< 0.001
4	1.36	1.22	1.51	< 0.001	1.38	1.24	1.54	< 0.001
5	1.47	1.32	1.64	< 0.001	1.49	1.33	1.66	< 0.001
*Ethnicity*								
Malay	1.59	1.33	1.91	< 0.001	1.71	1.41	2.07	< 0.001
Chinese	1.03	0.86	1.23	0.787	1.10	0.91	1.33	0.327
Indian	1.00				1.00			
Other Bumiputeras	1.74	1.38	2.19	< 0.001	1.69	1.32	2.17	< 0.001
Others	1.58	1.13	2.23	0.008	1.63	1.16	2.30	0.006
*Consuming fast food*								
Less than 3 days per week	1.00				1.00			
At least 3 days per week	1.88	1.69	2.08	< 0.001	1.73	1.55	1.93	< 0.001
*Carbonated soft drinks consumption*								
Less than 1 time per day	1.00				1.00			
At least 1 time per day	1.50	1.40	1.60	< 0.001	1.59	1.48	1.70	< 0.001
*Fruits intake*								
Less than 2 servings per day	1.31	1.23	1.39	< 0.001	1.44	1.35	1.54	< 0.001
At least 2 servings per day	1.00				1.00			
*Vegetable intakes*								
Less than 3 servings per day	1.00				1.00			
At least 3 servings per day	0.88	0.82	0.95	< 0.001	1.03	0.95	1.12	0.499

## Discussion

This study uncovered a depression prevalence rate of 26.9% among secondary school students. This discovery indicates a lower prevalence of depression when compared to a study conducted by Normala et al., which involved 1800 Malaysian secondary school adolescents aged 13 to 17 years and reported a depression prevalence of 32.7% among adolescents. It is noteworthy that Normala's study used the same depression assessment tool, the PHQ-9, but was conducted on a more limited scale, focusing on 10 out of 37 randomly selected secondary schools in the Hulu Langat district of Selangor [21]. When compared to NHMS 2019, the prevalence in the current study was higher. However, it is essential to note that NHMS 2019 is a national household survey focusing on the entire Malaysian population, not specifically on adolescents [5]. Furthermore, when making a comparison between the prevalence of depression among Malaysian adolescents and Japanese adolescents, it's evident that Malaysia had a notably higher rate, standing at 26.9% compared to Japan's 11.1% [22]. Conversely, the results of our current study aligned with those from Bangladesh, where the prevalence of depression among adolescents was also reported to be 26.5% [23]. It's important to note that both the studies conducted in Japan and Bangladesh utilized the same assessment tool as the one employed in our current investigation, which is the PHQ-9. In conclusion, the variation in depression prevalence can be attributed to a multitude of factors, including demographic characteristics, methodological disparities, as well as cultural, geographical, socioeconomic, and socio-political influences. Understanding these factors is essential for designing effective health policies and programmes that can enhance the health and well-being of adolescents in diverse countries.

The outcomes derived from the multiple logistic regression analysis unveiled that female adolescent exhibited a higher susceptibility to encountering depression, aligning with findings from prior research studies [2, 24]. Conversely, earlier research has posited that the elevated prevalence of depression among adolescents might be associated with factors like inadequate parental care, where some parents exhibit stricter behaviours towards female adolescents when compared to their male counterparts. Additionally, there exists a prevalent notion of diminished expectations concerning the capabilities and accomplishments of female adolescents in comparison to males [25]. Moreover, gender and cultural backgrounds can play a role in these disparities. It has been observed that male adolescents often attempt to endure depression symptoms unless they reach an extremely severe state, whereas females tend to express their symptoms at earlier stages [26]. These multifaceted elements collectively contribute to the divergent experiences of depression between female and male adolescents.

In terms of ethnicity, our study revealed that Malays, Other Bumiputeras, and Others were more prone to experiencing depression compared to Indians. This finding contrasts with a previous study that had indicated a higher susceptibility to depression among Chinese and Indian individuals [2]. However, due to limited survey data, we were unable to conduct a more comprehensive examination of the impact of these factors.

Furthermore, our current study has illuminated that older adolescents are at a higher risk of experiencing depression, a trend consistent with the findings from a study conducted by Xu H et al. [27]. This implies that as adolescents progress in age, they confront an increased vulnerability to developing depressive symptoms. Several contributing factors may underlie this pattern, including the physical, emotional, and social transformations experienced during adolescence, amplified academic pressures, evolving family dynamics, and heightened emotional awareness among older teenagers [28–30]. A comprehensive understanding of these intricacies is essential for addressing the mental well-being of older adolescents.

Recent research findings have hinted at a possible connection between less healthy eating habits and depression [10, 11]. By shedding light on these modifiable factors, we can deepen our understanding and empower public health authorities, healthcare professionals, and individuals to more effectively address and alleviate depression.

The results of this study have unveiled a noteworthy correlation between unhealthy dietary behaviours, especially the consumption of fast food, and the presence of depression. These findings are in line with previous research, including prospective studies involving Iranian [31] and Korean adolescents [32]. The link between fast food consumption and depression can be attributed to several factors. Firstly, fast foods tend to be deficient in essential nutrients and are often characterized by elevated levels of saturated fats, refined sugars, and additives. This subpar nutritional profile may impact the normal functioning of the brain, particularly its role in neurotransmitter synthesis, including serotonin—an important neurotransmitter closely tied to mood regulation. Serotonin, often referred to as the "feel-good" neurotransmitter, plays a pivotal role in controlling mood, emotions, and overall well-being [33–35]. A deficiency in serotonin levels has been associated with the emergence of depressive symptoms. The inadequacy of key nutrients in fast foods, which are essential for supporting serotonin production, could disrupt the delicate balance of neurotransmitters in the brain. This disruption might contribute to negative mood states and potentially elevate the risk of depression [36, 37]. In essence, the observed association between fast food consumption and depression in this study underscores the significant impact of the nutritional quality of one's diet on mental well-being. This highlights the importance of making informed dietary choices to promote both physical and mental health.

The current study has unveiled a significant positive association between the intake of carbonated soft drinks and the presence of depression. This discovery aligns with prior research, including a study involving Norwegian adolescents that also identified a connection between soft drink consumption and mental health concerns [38]. Moreover, multiple earlier studies have investigated the cross-sectional relationship between high carbonated soft drink consumption and depression or depressive symptoms [39–42]. It's essential to acknowledge that sugar-sweetened beverages, such as carbonated soft drinks, fall under the category of beverages containing caloric sweeteners. Previous research has indicated a link between excessive consumption of sugary beverages and an elevated risk of depression [7, 31]. To shed light on a potential biological mechanism underlying this association, researchers have put forth a hypothesis. Refined sugar, often abundant in sweetened beverages, has been shown to inhibit the growth hormone known as brain-derived neurotrophic factor (BDNF). A decrease in the normal functioning of BDNF has been associated with depression [43, 44]. In simpler terms, excessive refined sugar intake, as commonly found in sugary beverages like carbonated soft drinks, may have an adverse effect on the production and function of BDNF, potentially contributing to the onset or worsening of depressive symptoms. In the light of these findings, there is a compelling case for implementing policies aimed at restricting the availability of sugary beverages within educational institutions, such as schools, as well as in community and recreational centre. Concurrently, promoting the consumption of water as an alternative beverage choice could offer substantial benefits to the overall health and well-being of adolescents.

Contrary to the commonly held belief that unhealthy eating habits lead to depression, previous research offers an alternative perspective, suggesting that depressive symptoms can play a role in influencing unhealthy eating behaviours. Earlier studies have found that adolescents experiencing symptoms of depression encounter greater difficulties in maintaining a healthy diet [13]. Additionally, an interesting observation is that adolescent girls may resort to snacks and junk food as a way of coping with their depressive symptoms [45]. A comprehensive study revealed a connection between poorer psychological health and a tendency to consume soda, sweetened drinks, French fries, and fast foods [46]. The emergence of depressive symptoms might steer individuals towards less healthy dietary choices, with emotional eating behaviour playing a crucial role as food becomes a means of regulating negative emotions [47]. Research explains that the consumption of sweet and fatty foods induces positive feelings and alleviates stress by affecting certain neurotransmitters like dopaminergic, along with enhancing the function of the serotonergic system [48]. As a result, individuals dealing with depression may show a preference for consuming foods high in sugar or fat, especially when facing perceived stress [48]. This intricate interplay highlights the complex relationship between mental health and dietary habits, emphasizing the need for a nuanced understanding when addressing these dynamics.

Similarly, our study has revealed a significant association between the adoption of healthy dietary habits, specifically the consumption of fruits, and the presence of depression. These findings align with previous research outcomes [15, 32]. This observed phenomenon may be attributed to the nutritional richness of fruits, as they are known for their diverse and abundant nutritional content. They serve as plentiful sources of dietary fibre, essential vitamins, and minerals [49], and they are also packed with dietary antioxidants [50]. These components collectively contribute to the nutritional value of fruits. Firstly, dietary fibre, which is abundant in fruits, plays a crucial role in maintaining digestive health. It aids in regulating blood sugar levels and ensures a consistent energy supply throughout the day, reducing the likelihood of energy fluctuations and mood swings. Additionally, dietary fibre supports a healthy gut microbiome, and emerging research suggests that the connection between the gut and the brain could have a significant impact on mood regulation [51]. Secondly, fruits are rich in essential vitamins and minerals that are fundamental for overall well-being. Some of these nutrients, such as B vitamins like folate and minerals like magnesium, have been associated with mood regulation. For instance, deficiencies in folate have been linked to an increased risk of depression. The presence of these nutrients in fruits may contribute to a more balanced and stable mood [52–54]. Lastly, the dietary antioxidants found in fruits are renowned for their ability to combat oxidative stress and inflammation in the body. Oxidative stress and inflammation have been implicated in the development of mood disorders, including depression [54]. Therefore, the antioxidant-rich nature of fruits may help mitigate these factors and exert a protective effect on mental well-being. In summary, the observed negative correlation between fruit consumption and depression in our study suggests that the nutritional richness of fruits, encompassing dietary fibre, essential vitamins, minerals, and dietary antioxidants, may collectively contribute to their potential in alleviating depressive symptoms. This underscores the significance of incorporating a variety of fruits into one's diet not only to promote physical health but also to support mental well-being. However, it's worth noting that in our study, we did not find a significant link between adolescents' vegetable consumption and depression, despite previous research findings suggesting such a connection [15, 16, 32].

Similar to any scientific inquiry, the current study presents both strengths and limitations that necessitate consideration. One of its notable strengths lies in the comprehensive and ethnically diverse composition of the study's participant pool. In essence, the large and ethnically varied sample size equips the study with the capability to detect meaningful relationships and associations within the data, thereby elevating the reliability of the findings. Furthermore, this study distinguishes itself by focusing on a population-based sample rather than a clinical one. This distinction holds particular significance because it broadens the study's sphere of relevance. By examining depressive symptoms and dietary behaviours within a broader, community-based sample of adolescents, the findings are more likely to have practical applicability in the real world. In other words, the insights gleaned from this research extend beyond a specific clinical context and can be more broadly applied to a larger population of adolescents.

However, it's essential to acknowledge a significant limitation inherent in the study's design, namely, its cross-sectional nature. A cross-sectional study collects data from participants at a single point in time, offering a snapshot of information. While cross-sectional studies are valuable for identifying associations and trends within a population, they are incapable of establishing causality. In other words, the study can reveal that certain dietary behaviours are linked to depressive symptoms, but it cannot definitively ascertain whether one directly causes the other. Additionally, all the information in the study was self-reported, introducing the possibility of information bias and recall bias that may impact the conclusions.

## Conclusions

One out of every four Malaysian adolescents was discovered to be experiencing depression. Factors associated with depression included gender (being female), higher grade levels, and belonging to Malay, Other Bumiputeras and Others ethnic backgrounds. Moreover, this study emphasizes strong cross-sectional evidence establishing a connection between healthy dietary habits, specifically consuming fewer than two servings of fruits per day, and unhealthy dietary behaviours such as frequent consumption of fast food (at least three days per week) and daily intake of carbonated soft drinks, in relation to adolescents' experiences of depression. These findings emphasize the need for effective public health programmes that prioritize mental health and encourage healthy eating habits in adolescents, potentially leading to improved mental well-being and overall health outcomes for this vulnerable population.

## Data Availability

The data that support the findings of this study are available from Ministry of Health Malaysia but restrictions apply to the availability of these data, which were used under license for the current study, and so are not publicly available. Data are, however, available from the authors upon reasonable request and with permission of Ministry of Health Malaysia.

## Abbreviations

AOR: Adjusted odd ratio
CI: Confidence interval
MREC: Medical Research and Ethics Committee
PHQ-9: Patient health questionnaire
PPS: Proportional to enrolment
SFAs: Saturated fatty acids
WHO: World Health Organization
